# MRI radiomics in head and neck cancer from reproducibility to combined approaches

**DOI:** 10.1038/s41598-024-60009-6

**Published:** 2024-04-24

**Authors:** Anna Corti, Stefano Cavalieri, Giuseppina Calareso, Davide Mattavelli, Marco Ravanelli, Tito Poli, Lisa Licitra, Valentina D. A. Corino, Luca Mainardi

**Affiliations:** 1 Department of Electronics, Information and Bioengineering, Politecnico di Milano, Via Ponzio 34/5, 20133 Milan, Italy; 2 Head and Neck Medical Oncology Department, Fondazione IRCCS, Istituto Nazionale dei Tumori, Milan, Italy; 3 Department of Oncology and Hemato-Oncology, Università degli studi di Milano, Milan, Italy; 4 Radiology Department, Fondazione IRCCS, Istituto Nazionale dei Tumori, Milan, Italy; 5 Unit of Otorhinolaryngology-Head and Neck Surgery, Department of Medical and Surgical Specialties, Radiological Sciences, and Public Health, ASST Spedali Civili of Brescia, University of Brescia, Brescia, Italy; 6 Unit of Radiology, Department of Medical and Surgical Specialties, Radiological Sciences, and Public Health, ASST Spedali Civili of Brescia, University of Brescia, Brescia, Italy; 7 Maxillo-Facial Surgery Division, Head and Neck Department, University Hospital of Parma, Parma, Italy; 8 Cardiotech Lab, Centro Cardiologico Monzino IRCCS, Milan, Italy

**Keywords:** Magnetic resonance imaging, Head and neck squamous cell carcinoma, Radiomic features, Prognostic models, Overall survival, Cluster analysis, Cancer imaging, Oncology, Prognostic markers, Head and neck cancer, Tumour biomarkers, Biomedical engineering, Data processing, Image processing, Computer science

## Abstract

The clinical applicability of radiomics in oncology depends on its transferability to real-world settings. However, the absence of standardized radiomics pipelines combined with methodological variability and insufficient reporting may hamper the reproducibility of radiomic analyses, impeding its translation to clinics. This study aimed to identify and replicate published, reproducible radiomic signatures based on magnetic resonance imaging (MRI), for prognosis of overall survival in head and neck squamous cell carcinoma (HNSCC) patients. Seven signatures were identified and reproduced on 58 HNSCC patients from the DB2Decide Project. The analysis focused on: assessing the signatures’ reproducibility and replicating them by addressing the insufficient reporting; evaluating their relationship and performances; and proposing a cluster-based approach to combine radiomic signatures, enhancing the prognostic performance. The analysis revealed key insights: (1) despite the signatures were based on different features, high correlations among signatures and features suggested consistency in the description of lesion properties; (2) although the uncertainties in reproducing the signatures, they exhibited a moderate prognostic capability on an external dataset; (3) clustering approaches improved prognostic performance compared to individual signatures. Thus, transparent methodology not only facilitates replication on external datasets but also advances the field, refining prognostic models for potential personalized medicine applications.

## Introduction

Head and neck squamous cell carcinoma (HNSCC) represents the seventh most common and the sixth most deadly tumor worldwide, accounting for over 800,000 new annual cases and more than 350,000 annual deaths^1^. HNSCC comprises a group of highly heterogeneous malignancies, arising from the mucosa of oral cavity, pharynx and larynx^2^. Nowadays, the tumor-node-metastasis (TNM) staging system is the main factor guiding risk assessment, treatment choice and prognosis, and it is based on the clinical, radiological and pathological assessment^3,4^. However, the low stratification performance of staging-based system, combined with the high heterogeneity of HNSCC and the emergence of personalized medicine, fostered the development of additional biomarkers to improve patient stratification and consequently identify tailored treatment decisions.

Radiomics refers to the quantitative extraction of high-throughput features from medical images combined with their mining and analysis through machine learning algorithms. Radiomic features provide information about the primary tumor and/or lymph nodes morphological and textural heterogeneity characteristics, offering a potential source of pre-operative, non-invasive and comprehensive image-based diagnostic and prognostic biomarkers^5^. The number of radiomic studies in the oncological field has dramatically increased in the last decade, from around 30 studies indexed in PubMed in 2015, to nearly 2000 in the year 2022 alone.

To date, the application of radiomics to HNSCC patients is gaining increasing interest, encompassing, among others, tumor characterization, diagnostic differentiation, molecular markers prediction, recurrence, treatment response and survival prognostication, as extensively reviewed elsewhere^6–9^. However, the generalizability and reproducibility of radiomic studies remains an open issue, impairing the clinical translation of radiomics^10^. Indeed, different sources of variabilities arise from the image acquisition scanners and parameters, to the pre-processing processes, the manual/semi-automatic segmentation, up to features extraction. Moreover, the lack of a common consensus in the radiomics methodology, combined with shortcomings in transparently reporting the study design and the methodological details make the reproduction of published findings and the implementation of the published prognostic model on external datasets challenging.

So far, various initiatives have been undertaken to promote the development and establishment of standardized and widely applicable radiomics methodologies^10,11^. These efforts include aspects regarding features standardization, as that provided by the “Image Biomarker Standardization Initiative”^12^, guidelines for reporting prognostic models, as demonstrated by TRIPOD (transparent reporting of a multivariable prediction model for individual prognosis or diagnosis)^13^, data sharing, as exemplified by “The Cancer Imaging Archive” (TCIA) platform^14^ and the development of the Radiomics Quality Score (RQS) to assess the quality of radiomic studies^15^. Despite the progresses made in this direction, the adherence of the studies to the aforementioned criteria remains low, as demonstrated by a recent work in which 77 radiomic studies in oncological field were evaluated according to the RQS and TRIPOD criteria and reported a mean RQS of 9.40 (out of the ideal score of 36) and a mean adherence rate for TRIPOD of 57.8%^16^. Reproducibility of radiomic analyses still remains an ongoing concern, hampering its effective translation in the clinical practice.

In this context, the aims of the present study were to (1) assess 7 published, reproducible prognostic radiomic signatures for the prognosis of overall survival in HNSCC patients, (2) evaluate their relationship and performances on an external dataset and (3) propose combined radiomic approaches to assess additive value of integrating single radiomic signatures. A common external dataset of HNSCC patients collected during the BD2Decide project^17^ and presenting with pre-treatment magnetic resonance imaging (MRI) images, was considered, thus restricting the applicability of the analysis to MRI-based radiomic studies.

## Materials and methods

**Figure 1:**
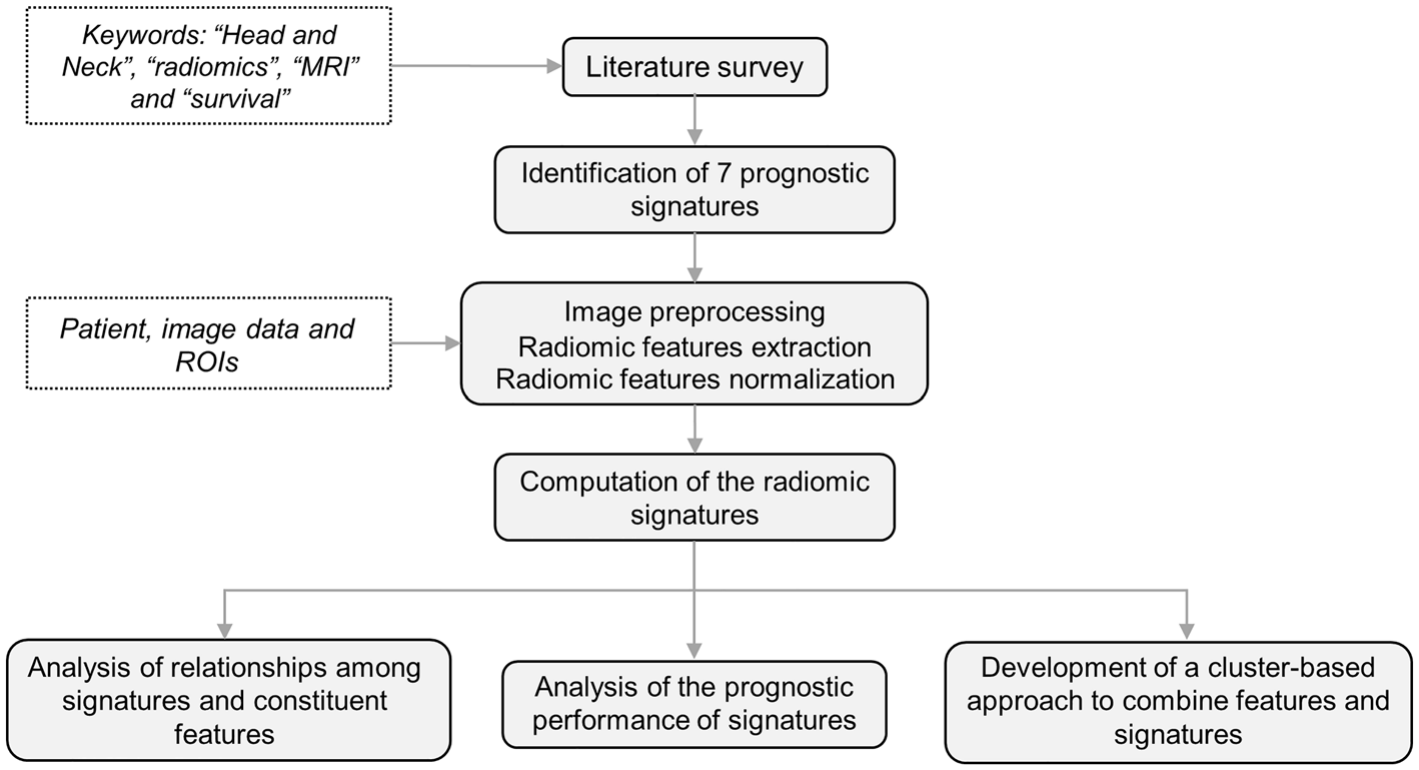
Workflow of the study.

Figure 1 outlines the workflow of the study, with details provided in subsequent sections. In summary, a literature review was conducted to identify reproducible MRI-radiomic prognostic signatures for overall survival in HNSCC. The methodologies reported in the literature were then applied to compute these signatures on the dataset under consideration. After image segmentation, specific image pre-processing techniques were employed to extract the relevant features for each signature, which were subsequently normalized. Following the computation of the radiomic signatures, analyses were performed to explore the relationships among the signatures and their constituent features, evaluate the prognostic performance of the signatures, and develop a combined approach to investigate whether integrating radiomic signatures or features could enhance performance.

### Radiomic signatures survey

A literature survey was performed to retrieve reproducible MRI-radiomic prognostic signatures for overall survival in HNSCC patients and compute the radiomic scores on our HNSCC dataset. To be strictly reproducible on an external dataset, a published radiomic signature must be provided with the following details: (1) the image pre-processing methods, (2) the list of the constitutive features, (3) the corresponding coefficients, (4) the operations performed on the features (e.g., details on the standardization process), and (5) the threshold adopted for the signature dichotomization, to evaluate the low/high risk stratification performance of the signature. Public database such as “Pubmed” (www.ncbi.nlm.nih.gov/pubmed) was considered, by imposing a selection using keywords as “Head and neck”, “radiomics”, “MRI” and “survival”. Search was extended from 2015 to today. Twenty-one papers were found and carefully analyzed to identify which of the above mentioned details were reported. As minimum requirement, publications should have included the list of features and the corresponding coefficients of the radiomic signature.

### Analyzed dataset

In the present study, a subset of HNSCC patients of the BD2Decide project (NCT02832102) presenting with pre-contrast T1-weighted (T1w) and T2-weighted (T2w) and post-contrast T1w (T1wCont) MR image sequence (acquired with 1.5 T scanner and with a 3 mm slice thickness) was considered^17^. To avoid overlap with the training dataset of previously developed signatures^18,19^, only prospective patients were included, leading to a subset of n = 58 patients. Table 1 summarizes the baseline clinical information of the selected patients included in the study. Patient data were collected from three participating centers: 33 patients from the Azienda Ospedaliero Universitaria di Parma (Italy), 21 patients from the Spedali Civili di Brescia (Italy) and 4 patients from the Istituto Nazionale dei Tumori (Italy).

**Table 1:** Clinical data of the patients used for the study.

Patient characteristics	Dataset N = 58
Date of diagnosis	2014–2017
Median follow-up	28.45 months (IQR 24.41—36.25)
Primary tumor subsite	
Oral cavity	49 (84%)
Oropharynx	4 (7%)
Larynx	4 (7%)
Hypopharynx	1 (2%)
Gender	
M	37 (64%)
F	21 (36%)
Median age	62 years (IQR 51–73.5)
cTNM 8th edition	
III	12 (21%)
IVa/b	46 (79%)
Smoking status	
Current/Former	27 (47%)
Never	14 (24%)
Unknown	17 (29%)
Survival status	
Alive	46 (79%)
Deceased	12 (21%)

### Image data acquisition and segmentation

T1w, T2w and T1wCont MRI were acquired using scanners with a field strength of 1.5 T and a turbo spin-echo pulse sequence. The contouring of the gross tumor volume was performed at the clinical centers using a semi-automatic segmentation software based on coupled shape modeling^20^. The region of interest (ROI), corresponding to the primary tumor, was segmented manually slice by slice by HNSCC expert radiologists (one for each center). T2w sequence was considered as reference to segment the ROI, and the other sequences (T1w an T1wCont) were used to check and correct the segmentations.

### Image pre-processing and features extraction

MRI images were pre-processed considering the methods declared in the original radiomic studies, which included some or all of the following steps: (1) denoising, through a 3D Gaussian filter with a 3 × 3 × 3 voxel kernel and σ = 0.5; (2) intensity non-uniformities correction, through the N4ITK algorithm^21^; (3) intensity standardization, using Z-score; (4) voxel size resampling to a specific isotropic resolution, through B-spline interpolation^22^, and (5) fixed-bin histogram discretization, with a specific number of bins. In case the image pre-processing methods were not mentioned, the default settings of Pyradiomics 2.2.0 software (open-source, available at https://github.com/Radiomics/pyradiomics and run on Python, used to extract the radiomic features) were considered, namely a fixed-bin histogram discretization, with 25 bins.

Radiomic features were extracted from the original image and transformed images, including the Laplacian of Gaussian (σ = 0.5, 1.0, 1.5, 2.0 and 5.0 mm) the wavelet, the square, the square root and the logarithm filters^23^. For each original and transformed image, features belonging to first order statistics, shape and size (only for original images), grey level co-occurrence matrix, grey level size zone matrix, neighboring gray tone difference matrix and grey level dependence matrix were extracted, for a total of n = 5064 features. Pyradiomics 2.2.0 software was used to extract the features^24^. If specified, features were normalized according to the reported methods. In case the details on the methods adopted to normalize the features were lacking, the following criteria were applied: (1) if the information was missing, the original features were considered, (2) if features normalization was mentioned, but without providing additional details, Z-score normalization was applied on our features.

### Radiomic signature computation, analysis, testing and combination

The identified radiomic prognostic signatures were computed for each patient of the dataset as the linear combination of the features and the corresponding regression coefficients. In case the threshold for the signature dichotomization was not provided, the median value of the radiomic signature on the present data was used to evaluate the low/high risk stratification performance of the signature. The relationship among the radiomic signatures was assessed by evaluating their correlation as well as the correlation among their features. The Spearman’s correlation coefficient was computed between: (1) each pair of signatures, and (2) each pair of constituent features. Moreover, a clustering analysis (through hierarchical clustering) was performed to identify clusters of highly correlated (both positively and negatively) features. Subsequently, the resulting correlation patterns and relationship among the clusters of features were evaluated by analyzing the types and meaning of the features composing each cluster.

The performance of the radiomic signatures was evaluated through the Kaplan–Meier curves^25^ for high- and low-risk groups with the associated p-value of the log-rank test^26^, Harrel’s concordance index (C-index) between the signature and the overall survival^27^, and the hazard ratio (HR).

To assess whether the combination of the signatures or their constitutive features could provide additive prognostic information compared to the single radiomic models, a cluster-based approach was considered. In particular, we tested the hypothesis that clusters of patients, grouped according to either the radiomic features (composing the signatures) or the signatures, presented significantly different overall survival. K-medoids clustering^28^ was adopted to generate feature-based and signature-based clusters of patients. The capability of the two clusters to stratify low- and high-risk patients was assessed by evaluating the Kaplan–Meier curves and the associated log-rank test p-value. Finally, the performance of the cluster-based approaches was compared with the single radiomic models: if a better stratification performance was found, this would demonstrate (1) a strong relationship among the developed radiomic signatures, in turn confirming their validity, (2) the potential of combining signatures in enhancing the predictive power of radiomic models and (3) the importance of good reproducibility of radiomic studies to contribute to advancements in the field.

### Ethics approval and consent to participate

The protocols were approved by the Ethical Committees of the participating centers and data acquisition followed the General Data Protection Regulation of the EU. Consent was obtained from all participants and/or their legal guardians. All methods were carried out in accordance with relevant guidelines and regulations and the study has been performed in accordance with the Declaration of Helsinki.

## Results

### Radiomic signatures

From the literature survey, 7 reproducible MRI-based radiomic signatures for HNSCC patients were identified^18,19,29–33^ as those which satisfy the minimum requirements for reproducibility. They are reported in Table 2, along with their constitutive features in Table 3. Five monomodal signatures were reported, with three of them based on features extracted from the T1wCont sequence (R1, R2 and R4) and the remaining ones from the T2w sequence (R5 and R7). R3 and R6 are multimodal radiomic signatures, with R3 based on T1w, T1wCont and T2w sequences and R6 on T1w and T2w sequences. Overall, 34 prognostic features (21 from T1wCont, 12 from T2w and 1 from T1w) were identified, with one feature (*T2w_waveletLLL_firstorder_Range*) selected in both R6 and R7. Specifically, (1) among the 21 T1wCont features, 13 were extracted from the wavelet transformation (textural features), 5 from the Laplacian of Gaussian transformation (3 first order and 2 textural features) and 3 from the original image (2 shape and 1 textural features); (2) among the 12 T2w features, 9 were extracted from the wavelet transformation (3 first order and 6 textural features), one from the Laplacian of Gaussian transformation (first order feature) and 2 from the original image (shape feature) and (3) the T1w feature was extracted from the original image (textural feature).

**Table 2:** Reported methodologies on the radiomic signature pipeline.

Sig.	Ref.	Image pre-processing	Features	Feature normalization	Signature threshold
R1	Bos 2021^29^	(iii); (iv); (v)	10 (T1wCont)	Z-score (no details)	NA
R2	Chen 2022^30^	NA	6 (T1wCont)	NA	NA
R3	Alfieri 2022^31^	(i); (ii); (iii); (iv); (v)	3 (T1w, T1wCont, T2w)	Z-score (µ and σ provided)	NA
R4	Siow 2022^32^	(ii); (iii); (iv); (v)	4 (T1wCont)	NA	0.5
R5	Mossinelli 2023^33^	NA	2 (T2w)	Unspecified standardization	NA
R6	Bologna 2023^19^	(i); (ii); (iii); (iv); (v)	4 (T1w, T2w)	Z-score (µ and σ provided)	NA
R7	Corti 2023^18^	(iii); (iv); (v)	5 (T2w)	Z-score (µ and σ provided)	0.082

(i) denoising, through a 3D Gaussian filter with a 3 × 3x3 voxel kernel and σ = 0.5; (ii) intensity non-uniformities correction, through the N4ITK algorithm; (iii) intensity standardization, using Z-score; (iv) voxel size resampling to a specific isotropic resolution, through B-spline interpolation, and (v) fixed-bin histogram discretization, with a specific number of bins. µ: media value for Z-score standardization σ: standard deviation for Z-score standardization. Unspecified standardization: the method for feature standardization is not known. NA: not available.

**Table 3:** Radiomic signatures with corresponding features.

Signature		Features
R1	*R1-1*	*T1wCont_waveletHLL_gldm_SmallDependenceEmphasis*
	*R1-2*	*T1wCont_waveletLLH_ngtdm_Busyness*
	*R1-3*	*T1wCont_waveletLLL_ngtdm_Busyness*
	*R1-4*	*T1wCont_waveletHHH_glszm_ZoneVariance*
	*R1-5*	*T1wCont_logsigma20mm3D_glcm_DifferenceVariance*
	*R1-6*	*T1wCont_waveletHHH_glszm_LargeAreaHighGrayLevelEmphasis*
	*R1-7*	*T1wCont_waveletHHH_ngtdm_Strength*
	*R1-8*	*T1wCont_waveletLHH_ngtdm_Complexity*
	*R1-9*	*T1wCont_waveletLHH_glcm_Correlation*
	*R1-10*	*T1wCont_logsigma20mm3D_glcm_InverseVariance*
R2	*R2-1*	*T1wCont_original_shape_Maximum3DDiameter*
	*R2-2*	*T1wCont_original_shape_Compactness1*
	*R2-3*	*T1wCont_original_glrlm_RunLengthNonUniformityNormalized*
	*R2-4*	*T1wCont_waveletHLL_glrlm_LongRunEmphasis*
	*R2-5*	*T1wCont_waveletLHL_glcm_JointEntropy*
	*R2-6*	*T1wCont_waveletHLH_glrlm_ShortRunHighGrayLevelEmphasis*
R3	*R3-1*	*T1w_original_glszm_SizeZoneNonUniformity*
	*R3-2*	*T1wCont_waveletLLL_ngtdm_Complexity*
	*R3-3*	*T2w_waveletHLL_gldm_DependenceVariance*
R4	*R4-1*	*T1wCont_logsigma15mm3D_firstorder_90Percentile*
	*R4-2*	*T1wCont_logsigma10mm3D_firstorder_Energy*
	*R4-3*	*T1wCont_logsigma10mm3D_firstorder_TotalEnergy*
	*R4-4*	*T1wCont_waveletLHL_glszm_SizeZoneNonUniformity*
R5	*R5-1*	*T2w_original_shape_Maximum2DDiameterRow*
	*R5-2*	*T2w_logsigma50mm3D_firstorder_Maximum*
R6	*R6-1*	*T1w_waveletLHL_firstorder_90Percentile*
	*R6-2*	*T2w_original_shape_VoxelVolume*
	*R6-3*	*T2w_waveletHHL_glrlm_GrayLevelNonUniformityNormalized*
	*R6-4*	*T2w_waveletLLL_firstorder_InterquartileRange*
	*R6-5*	*T2w_waveletLLL_firstorder_Range*
R7	*R7-1*	*T2w_waveletLLL_glrlm_LongRunEmphasis*
	*R7-2*	*T2w_waveletLLL_glrlm_RunVariance*
	*R7-3*	*T2w_waveletLLL_glrlm_RunPercentage*
	*R7-4*	*T2w_waveletLLL_firstorder_Range*
	*R7-5*	*T2w_waveletLLL_glrlm_ShortRunEmphasis*

To reproduce R1, following the image pre-processing steps reported in the study, the features were normalized with Z-score standardization and the signature was dichotomized considering the median value. R2 was computed by applying the default Pyradiomics image pre-processing and by considering the original (not-normalized) features. Moreover, the median value of the signature was used as threshold for dichotomization. As regards R3, all the methods for image pre-processing and features normalization were reported in the study, and the signature was dichotomized based on the median value. R4 was reproduced by considering the reported pre-processing steps and the original features, with the declared dichotomization threshold. To reproduce R5, the default Pyradiomics image pre-processing steps were applied, features were normalized with Z-score standardization and the median value of the signature was considered for dichotomization. As regards R6, all the methods for image pre-processing and features normalization were reported in the study, and the signature was dichotomized based on the median value. Finally, R7 was computed by following the methods detailed in the study.

### Radiomic signature relationship

**Figure 2:**
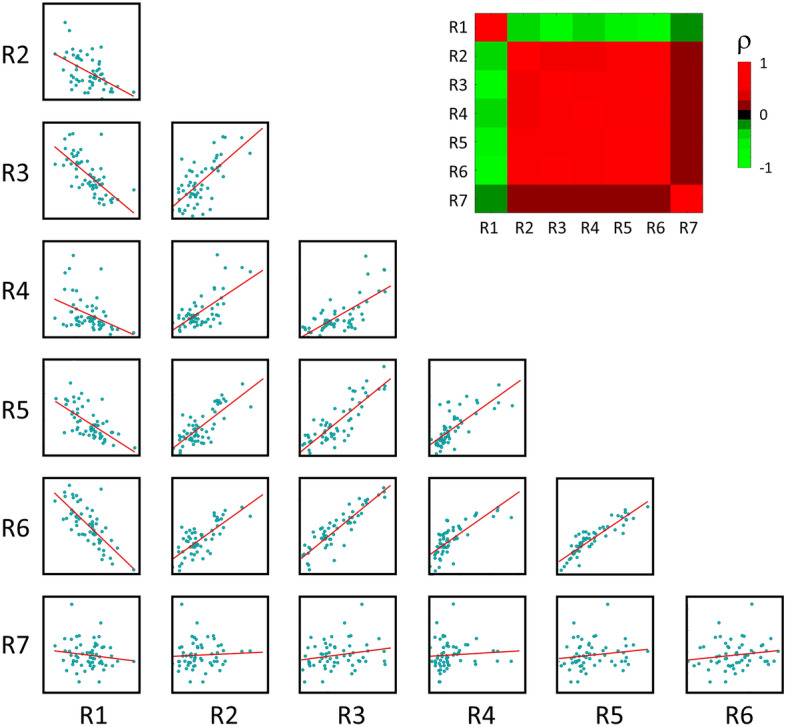
Correlation analysis on the radiomic signatures. Paired scatter plots between radiomic signatures and correlation matrix heatmap of Spearman’s correlation coefficients, computed between each pair of radiomic signature.

**Figure 3:**
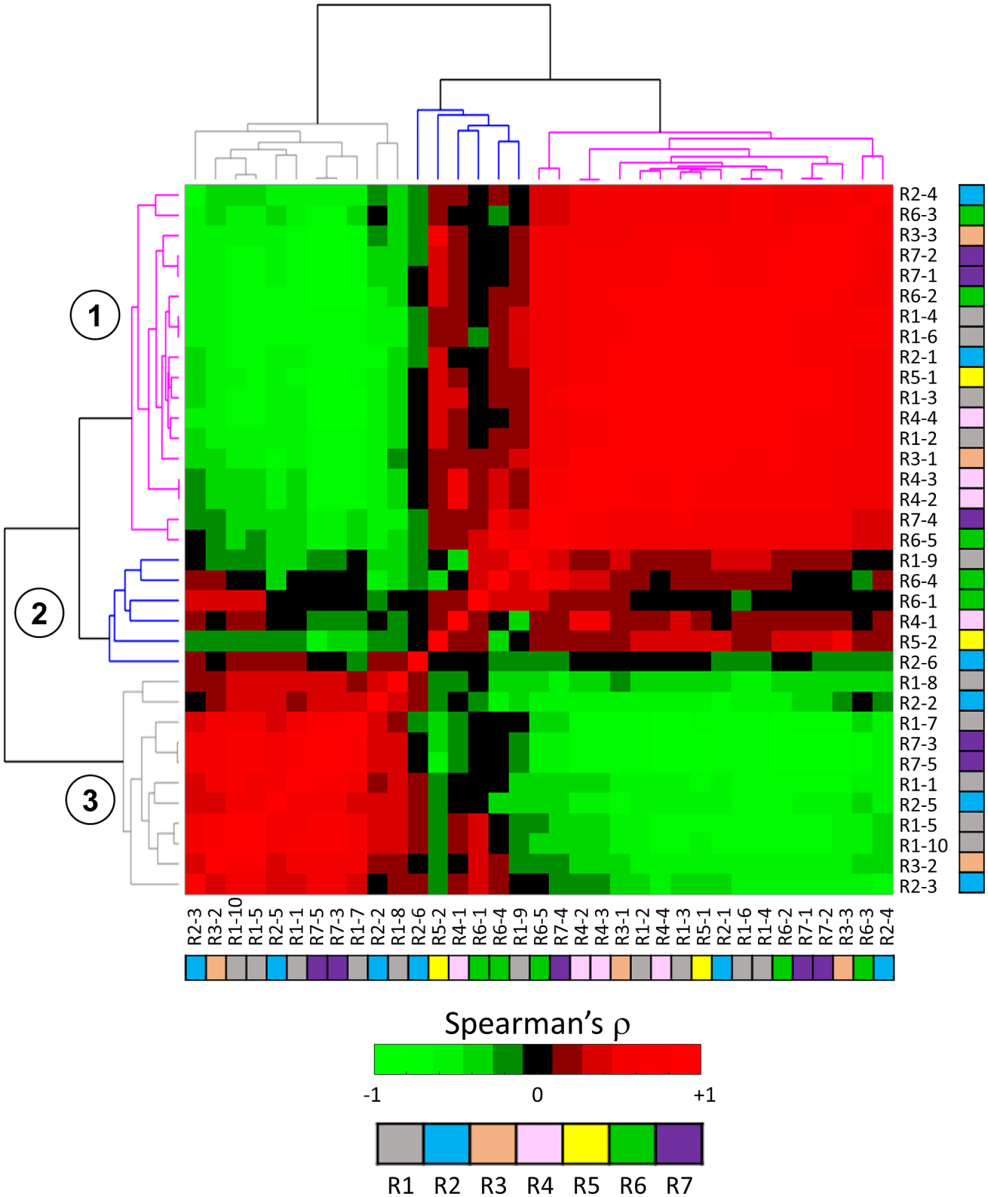
Correlation and clustering analysis on the radiomic features. Correlation-driven clustering and dendrogram of the 35 radiomic features. The radiomic features are labelled as the corresponding signature as reported in Table 3. Moreover, features are colored according to the radiomic signatures: R1 grey, R2 light blue, R3 orange, R4 pink, R5 yellow, R6 green and R7 violet. Spearman’s correlation coefficient was computed between each pair of radiomic feature.

The radiomic signatures exhibit strong correlations (with the exception of R7) among each other, as illustrated in Fig. 2. In particular, R1 demonstrates a negative correlation with all the other signatures (with Spearman’s ρ = − 0.72 between R1 and R3 and ρ = − 0.74 between R1 and R6), with the remaining signatures being positively correlated with each other (with high Spearman’s ρ of 0.77 between R3 and R5, of 0.86 between R3 and R6, of 0.75 between R4 and R5, of 0.77 between R4 and R6 and of 0.90 between R5 and R6). To further explore the interplay among the signatures, a clustering analysis on their constituent features was conducted. Figure 3 illustrates a hierarchical clustering based on Spearman’s correlation coefficient, calculated between each pair of radiomic features, aiming to uncover the relationships among the 35 features comprising the 7 radiomic signatures. This analysis unveiled three distinct clusters of features, two of them exhibiting specific correlation patterns. Notably, the first and third clusters (depicted by purple and grey trees on the left-axis of Fig. 3, respectively) showed a high inverse correlation: they comprise features that are highly positively correlated within their respective clusters but inversely correlated with features from the other cluster. In the first cluster, 11 out of 18 features, and in the third cluster, 10 out of 11 features are textural and pertain to aspects such as the heterogeneity of grey level zones, busyness, complexity of the images, and the distribution of grey level runs within the ROI. As expected, strong positive correlations are evident among features within the first cluster. Examples include R2-4 with R7-1 measuring the distribution of long run lengths (*glrlm LongRunEmphasis),* or R1-3 with R1-2, characterizing the change from a pixel to its neighbor (*ngtdm Busyness)*, and R3-1 with R4-4, both describing the variability of size zone volumes in the ROI (*glszm SizeZoneNonUniformity)*. Similar findings apply to the third cluster, such as the relationship between R7-3 and R7-5, both linked to the presence of short runs (*glrlm RunPercentage and ShortRunEmphasis)* and R3-2 and R1-8 (*ngtdm Complexity*), characterizing the primitive components in the image. Furthermore, there is a strong negative correlation between the first and the third clusters, as evidenced by the relationship between R1-3/R1-2 (cluster 1, *ngtdm Busyness*) and R1-7 (cluster 3, *ngtdm Strength)*. The former are associated with a rapid change of intensity between pixels and neighbors, while the latter is linked to a slow change. Similarly, R7-1/R2-4 (cluster 1, *glrlm LongRunEmphasis*) and R7-3/R7-5 (cluster 3, *glrlm RunPercentage and ShortRunEmphasis*) exhibit negative correlations, with the former associated with longer run lengths, and the latter with shorter run lengths. As for the second cluster (blue), it mainly comprises first-order features (4 out of 6 features), displaying predominantly positive correlations with the first cluster, and negative correlations with the third cluster. Notably, high positive and negative correlations are evident not only among features from different signatures but also within the same signature. For example, R1 is composed by 4 features belonging to the first cluster and 5 features belonging to the third cluster, resulting in high absolute correlations. Similarly, R7 presents 3 features from the first cluster, and 2 from the third cluster. These findings align with the fact that the feature selection processes used for developing both R1 and R7 did not consider a criterion based on correlations. This challenges the common belief that effective prognostic signatures should avoid the inclusion of correlated features.

### Radiomic signature testing

**Figure 4:**
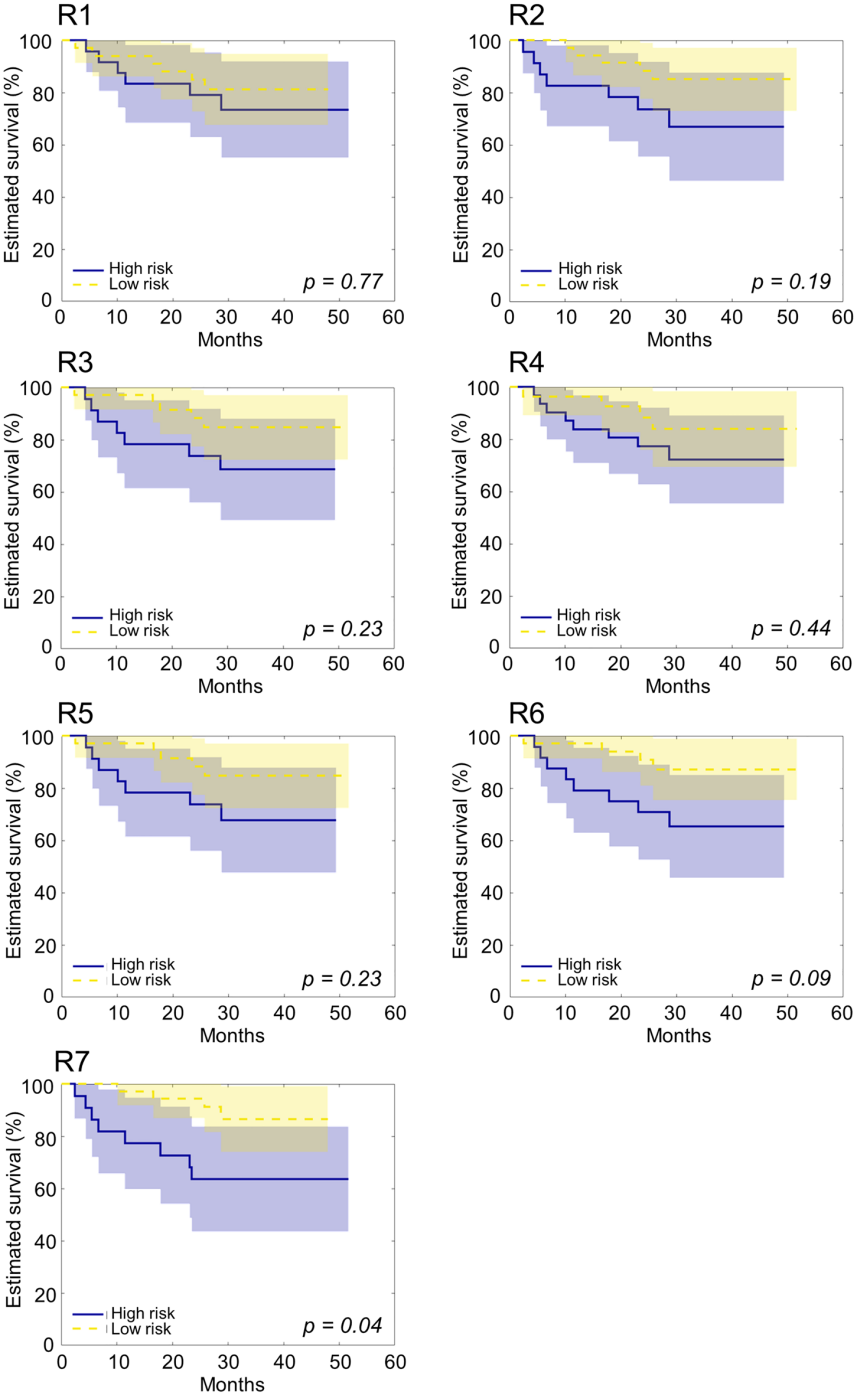
Kaplan–Meier curves of the low-risk (in yellow) and high-risk (in blue) patient groups according to the stratification obtained by the reproduced radiomic signatures (R1 to R7) on the dataset of n = 58 patients. Shadows represent the 95% confidence interval. The p-value of the log-rank test is also provided.

Figure 4 shows the Kaplan-Maier curves for the high- and low-risk groups according to the stratification obtained by radiomic signatures, with Table 4 detailing the corresponding C-index, HR and log-rank p-value. With the exception of R4, all the signatures presented C-index > 0.6, with R2, R3, R5, R6 and R7 HR > 2. However, only R7 significantly stratified low–high risk patients, providing the best performance, with median C-index 0.74, HR 4.24 and log-rank p = 0.04. Despite the different performances, the signatures present similar Kaplan-Maier curves, particularly R2, R3, R5 and R6. Moreover, it is important to highlight that R5 and R7 were specifically tailored for patients with oral cavity squamous cell carcinoma, which represented the most prevalent tumor location in the dataset under consideration. Additionally, R3 and R6 were developed for patients with HNSCC, with a substantial proportion being oral cavity patients. In contrast, R1 was designed specifically for oropharyngeal cancer, while R2 and R4 were focused on hypopharyngeal cancer, which constituted only 7% and 2% of the dataset, respectively. Finally, as shown in the supplementary material, R2 and R5 demonstrated different performances when different image pre-processing methods were applied. In particular, R2 was associated with a C-index varying between 0.61 and 0.64 and an HR varying between 1.22 and 2.62, while R5 was associated with a C-index varying between 0.60 and 0.64 and HR varying between 2.46 and 3.36.

**Table 4:** Radiomic signatures prognostic performance.

Signature	C-index	Log-rank HR	Log-rank *p*
R1	0.63 [IQR 0.56 0.69]	1.41 [95% CI 0.45 4.44]	0.77
R2	0.63 [IQR 0.59 0.69]	2.62 [95% CI 0.81 8.53]	0.19
R3	0.61 [IQR 0.56 0.68]	2.43 [95% CI 0.75 7.81]	0.23
R4	0.59 [IQR 0.52 0.63]	1.84 [95% CI 0.59 5.71]	0.44
R5	0.61 [IQR 0.55 0.67]	2.46 [95% CI 0.76 7.95]	0.23
R6	0.62 [IQR 0.56 0.67]	3.27 [95% CI 1.02 10.45]	0.09
R7	0.74 [IQR 0.69 0.78]	4.24 [95% CI 1.28 13.99]	0.04
Feature-based cluster	NA	4.51 [95% CI 1.28 15.91]	0.04
Signature-based cluster	NA	7.58 [95% CI 1.79 32.15]	0.02

C-index, Harrel’s concordance index; HR, hazard ratio; NA, not available.

### Radiomic signature combination

**Figure 5:**
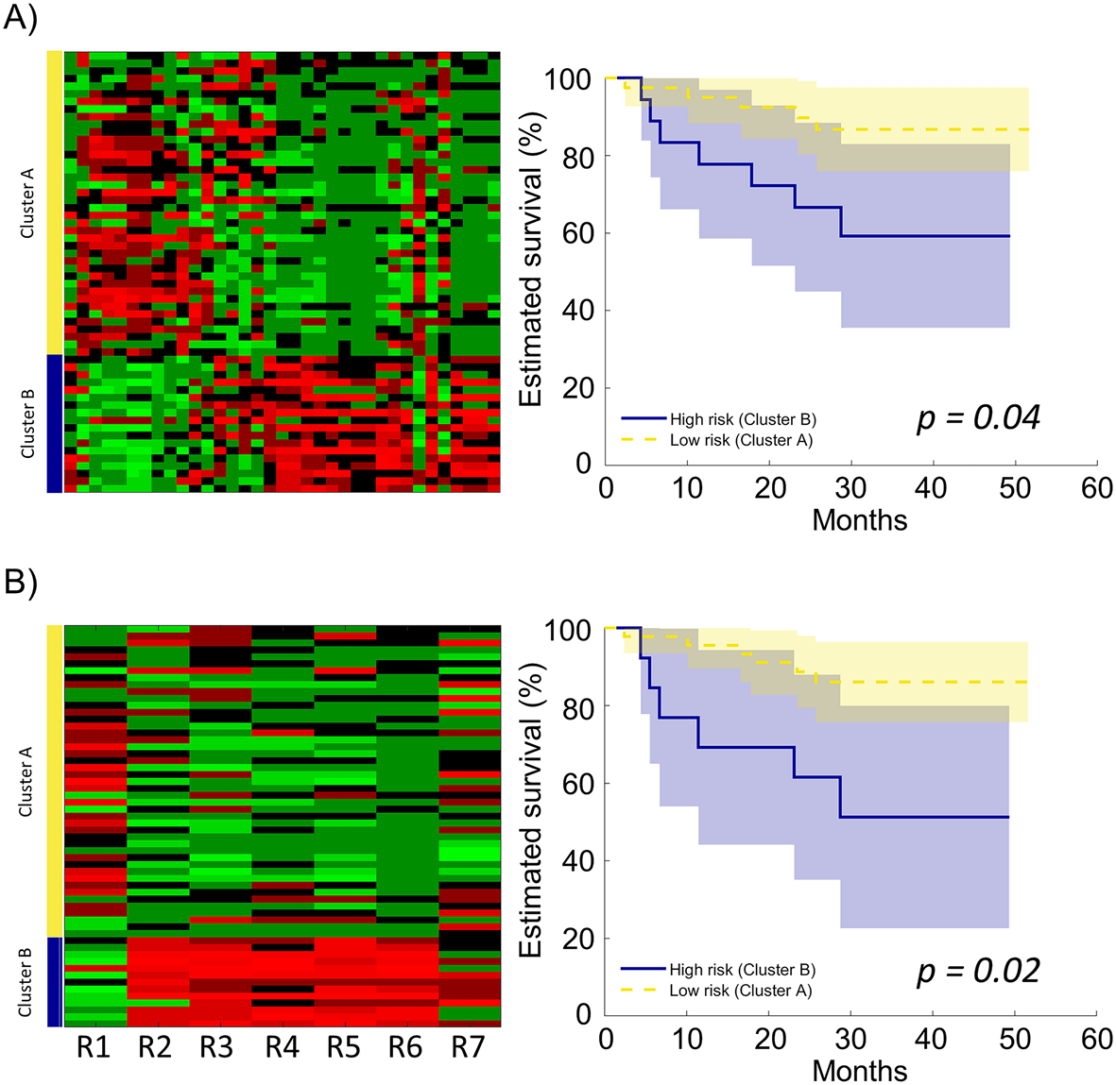
(**A**) Left: patient clustering (Cluster A and Cluster B) based on the 35 radiomic features. Right: Kaplan–Meier curves for the feature-based radiomic clusters. (**B**) Left: patient clustering (Cluster A and Cluster B) based on the radiomic signatures (R1–R7). Right: Kaplan–Meier curves for the signature-based radiomic clusters.

Figure 5 shows the feature-based (Fig. 5A) and the signature-based (Fig. 5B) patient clustering, generated through K-medoids algorithm. In particular, two clusters of patients (Cluster A in yellow, and Cluster B in blue) were identified based on the feature or signature values. In the feature-based case, Cluster A comprised 40 patients and Cluster B 18 patients, while in the signature-based case, Cluster A comprised 45 patients and Cluster B 13 patients. In both cases, Cluster A and Cluster B exhibit significant differences in overall survival, effectively stratifying patients into low- and high-risk groups (Fig. 5). Moreover, both cluster-based stratifications outperformed the single radiomic signatures, with a HR of 4.51 [95% CI 1.28 15.91] and log-rank p = 0.04 for the feature-based case, and a HR of 7.58 [95% CI 1.79 32.15] and log-rank p = 0.02 for the signature-based case (Table 4).

## Discussion

The increasing number of radiomic studies and their applications in oncological field promise a potential role in the emerging personalized medicine. However, the absence of a standardized radiomics pipeline combined with the significant variability associated with the methodology (e.g., image acquisition, pre-processing, segmentation, software for features extraction) and the lack of transparency in reporting methodological details and study design, hamper the reproducibility of the analysis and the replication of its results. This in turn impedes the potential translation of radiomics to clinics.

Herein, a literature of survey was performed in order to identify reproducible MRI-based radiomic signatures for prognosis of overall survival in HNSCC patients, according to five criteria, namely (1) details on image pre-processing, (2) list of features, (3) list of coefficients, (4) details on feature normalization and (5) value for signature dichotomization. Among the 21 MRI-based prognostic signatures identified for overall survival in HNSCC, only one satisfied all the specified criteria, highlighting the current challenge. Consequently, the analysis was extended to the 7 studies reporting, at minimum, the list of features and coefficients (criteria (2) and (3)), being essential for replicating the signature. Among the 7 studies, 5 reported details on image pre-processing steps, 3 on feature normalization and only 2 on the threshold for signature dichotomization. Consequently, with the exception of R7, the faithful reproduction of the signatures was not possible. This sheds light on the need for defining a common consensus about the transparency of the delivered information, which is required to correctly replicate the radiomic analyses and subsequently to lay the foundations for a potential clinical translation. However, assumptions were introduced to address the lack of details regarding image pre-processing, feature normalization and dichotomization and replicate the radiomic score on our dataset.

By reproducing the signatures on a common external dataset and by examining the correlations among them and their constituent features, the following key finding emerged. High absolute correlations were found among the signatures, and the correlation-based clustering on the inherent features revealed clusters containing features characterizing similar aspects of the ROI. This suggested that, despite the utilization of different features selection methods, the identified features, although different, describe similar aspects of the lesion. Indeed, most of the features were textural, demonstrating that tumor heterogeneity, which is a remarkable characteristic of HNSCC, potentially contains prognostic information. Moreover, certain signatures, notably R1 and R7, are built upon correlated features, indicating that avoiding a correlation-based feature selection approach may also be a reasonable strategy for developing prognostic signatures.

The prognostic efficacy of the radiomic signatures was also assessed. Notably, despite the uncertainties related to the assumptions introduced to reproduce the signatures, potentially affecting the observed outputs, the signatures demonstrated a moderate prognostic power even on an external dataset, exhibiting similar Kaplan–Meier curves, C-index and HR ranges. This indicates that, despite the varied methodologies employed in the 7 selected studies, they all resulted in the generation of similar signatures with comparable prognostic performances.

R7 emerged as the most performing signature, with statistically significant stratification performance. Except for R4, the other signatures demonstrated similar prognostic performances (C-index > 0.6) though without statistical significance. It is crucial to note that R7 was the only fully-reproducible signature. Consequently, the assumptions made to reproduce the other signatures likely influenced their performance, as demonstrated by the analysis of R2 and R5 under different image preprocessing methods. In addition, the stratification of high- and low-risk patients is inherently tied to the chosen threshold, introducing uncertainty that impacts results in terms of HR and Kaplan–Meier curves. Furthermore, the diminished performance of R1, R2 and R4, may also arise from their specificity for oropharyngeal and hypopharyngeal cancer patients, who are underrepresented in the dataset under consideration (predominantly comprising oral cavity cases).

The combination of the radiomic models through feature- and signature-based clustering approaches resulted in enhanced prognostic performance compared to the radiomic signatures alone. Notably, the signature-based cluster approach exhibited the most effective performance in patient stratification. This superiority can be attributed to the fact that the signatures already inherently embody an optimal combination of their constituent features. However, it is important to acknowledge that the signature-based cluster approach, reliant on signatures, necessitates the reporting of both features and coefficients. This approach is inherently more restrictive, in terms of reproducibility, compared to the feature-based cluster approach, which solely necessitates reporting constituent features. Consequently, the feature-based clustering approach holds the potential for broader applicability. Another advantage of the suggested cluster-based approach (either for the feature- or cluster-based case) is that it does not require a training-test procedure, thus being suitable for relatively small datasets. Overall, the improved performance achieved through the cluster-based approach underscores the importance of transparent and detailed reporting of the methodological steps. Such transparency not only facilitates the replication of signatures on external datasets, but also contributes to the continuous advancement of the field, paving the way for improved prognostic models with potential applications in the realm of personalized medicine. Towards this goal, in future studies, reproducible radiomic signatures or documented features could be integrated with gene expression signatures to enhance the prognosis of HNSCC. Notably, leveraging data from The Cancer Genome Atlas^34^, there has been a substantial effort in developing gene expression signatures for various anatomical subsites of HNSCC^35^. Subsequent research endeavors could concentrate on replicating both radiomic and gene expression signatures using an external dataset (provided that both image and microarray data are available), to evaluate the added value of incorporating image-based markers alongside biological markers. Moreover, other computational methods, such as graph convolutional network^36^ or theoretical models based on ordinary differential equations can be adopted to explore the interrelationships of radiomic features and biological markers^6,37,38^.

The present study is not exempt from limitations, which are mainly associated with the assumptions made for the computation of the radiomic signatures and the lack of homogeneity in the dataset across different tumor locations. In particular, as regards the assumptions introduced to address the insufficient details reporting, we have demonstrated how the selected image pre-processing methods affect the signature performances. In future, it would be interesting to explore also how different approaches on features standardization may impact on the results. Moreover, while herein the median value was used to dichotomize the signature (when the effective threshold was not provided), in future, if larger datasets are available, a partition of the data can be performed to optimize the signature threshold on the training set and use it to stratify the test set. In relation to our dataset, the absence of uniformity among tumor locations, particularly the imbalance towards the oral cavity location, may have influenced the observed results. Indeed, a dataset with more consistent tumor locations would have ensured a more uniform representation of R1, R2, and R4 signatures, which are specific to oropharyngeal and hypopharyngeal cancer patients, being the minority categories. To this aim, future analyses should either consider more heterogeneous datasets or focus only on signatures that were developed for the specific tumor locations of the considered dataset. Further investigation in future research could also explore alternative combination methodologies, such as fitting multivariate Cox proportional hazard regression models using the 7 radiomic signatures or their individual features as a basis. While the suggested cluster-based approach provides the advantage of not necessitating retraining, thus being suitable for relatively small datasets, this alternative method would require a training-test procedure, thus necessitating larger datasets.

Overall, although the literature includes several meta-analysis studies on radiomics^39–41^, to the best of the authors’ knowledge, the present study represents the initial endeavor to (1) replicate published radiomic signatures on an external dataset, offering a potential method to address the insufficient reporting, (2) provide a detailed characterization of the reproduced radiomic signatures, and (3) propose combined approaches to enhance the prognostic performance. The proposed study yielded to key findings. First, despite different methodologies were adopted in the radiomic signatures design, the 7 signatures and their features were highly correlated suggesting consistency in the identified features, being associated with similar lesion properties (mainly textural). Second, the signatures exhibited a moderate prognostic performance on an external dataset, despite the uncertainties related to their reproduction. Third, combining radiomic signatures through clustering approaches improved the prognostic performance compared to using individual radiomic signatures. Consequently, detailed methodological transparency not only aids replication on external datasets but also propels the field forward, enhancing prognostic models for potential applications in personalized medicine. Thus, the proposed approach has the potential to demonstrate the practical applicability of radiomic studies and facilitate their clinical translation.

## Conclusion

This study demonstrated the feasibility of replicating, testing and comparing published radiomic signatures on an external dataset, provided that sufficient methodological details are described. Moreover, a novel cluster-based approach was proposed to combine radiomic signatures and features, resulting in increased prognostic performance compared to the individual radiomic signatures. This not only underscores the advantages of transparently reporting details to advance radiomics for patient stratification but also provides a feasible and replicable approach which that can be utilized in forthcoming investigations to predict outcomes for new patients. Specifically, the feature-based clustering approach, which solely depends on feature values, is less reliant on the rigorous reproducibility of radiomic signatures, thus offering wider applicability.

Overall, future efforts should be put in reporting radiomic analyses in order to enable their full reproduction in view of their potential translation in clinics.

### Supplementary Information

Supplementary Information.

## Data Availability

The datasets generated during and/or analysed during the current study are available from the corresponding author on reasonable request.
