# Supplementary material for: MRI radiomics in head and neck cancer from reproducibility to combined approaches
Source: Sci Rep. 2024 Apr 24;14:9451. doi: 10.1038/s41598-024-60009-6 (PMC11043398; doi:10.1038/s41598-024-60009-6)
Supplement: Supplementary file 1 — Supplementary Information. [file 41598_2024_60009_MOESM1_ESM.pdf]

MRI Radiomics in Head and Neck Cancer from Reproducibility to Combined Approaches

Supplementary Material

Anna Corti[1,*], Stefano Cavalieri[2,3], Giuseppina Calareso[4], Davide Mattavelli[5], Marco Ravanelli[6], Tito Poli[7], Lisa Licitra[2,3], Valentina Corino[1,8], Luca Mainardi[1]

1. Department of Electronics, Information and Bioengineering, Politecnico di Milano, Milan, Italy
2. Head and Neck Medical Oncology Department, Fondazione IRCCS, Istituto Nazionale dei Tumori, Milan, Italy
3. Department of Oncology and Hemato-Oncology, Università degli studi di Milano, Milan, Italy
4. Radiology Department, Fondazione IRCCS, Istituto Nazionale dei Tumori, Milan, Italy
5. Unit of Otorhinolaryngology-Head and Neck Surgery; Department of Medical and Surgical Specialties, Radiological Sciences, and Public Health; ASST Spedali Civili of Brescia, University of Brescia, Brescia, Italy
6. Unit of Radiology; Department of Medical and Surgical Specialties, Radiological Sciences, and Public Health; ASST Spedali Civili of Brescia, University of Brescia, Brescia, Italy
7. Maxillo-Facial Surgery Division, Head and Neck Department, University Hospital of Parma, Parma, Italy
8. Cardiotech Lab, Centro Cardiologico Monzino IRCCS, Milan, Italy

***Address for correspondence:**

Anna Corti, PhD

Department of Electronics, Information and Bioengineering, Politecnico di Milano, Via Ponzio 34/5, 20133 Milan, Italy - anna.corti@polimi.it

Influence of the image preprocessing method

The default setting of Pyradiomics for image preprocessing includes a fixed-bin histogram discretization with 25 bins. Optionally, Z-score image standardization can be enabled, as well as voxel size resampling to a specific isotropic resolution (with B-spline interpolator as default option). To assess the impact of the image preprocessing method on the radiomic signature performance, 4 preprocessing settings, listed in Supplementary Table 1, were tested to reproduce R2 and R5, given that a preprocessing method was not declared in the studies. Supplementary Table 2 and Figure 1 provide the performance of R2 and R5 on overall survival prediction and patient high- low-risk stratification, at the 4 different preprocessing methods. It can be noted that the signatures performances are highly affected by the preprocessing method, in terms of C-index, HR, log-rank p-value and Kaplan-Meier curves.

Supplementary Table 1 Image preprocessing methods

Method	histogram discretization	Z-score	Resampling
A	25 bins	Disabled	Disabled
B	25 bins	Enabled	Disabled
C	25 bins	Disabled	Enabled, 3 mm isotropic resolution
D	25 bins	Enabled	Enabled, 3 mm isotropic resolution

Supplementary Table 2 Radiomic signatures prognostic performances at different image preprocessing methods

Signature	Preprocessing method	C-index	Log-rank HR	Log-rank *p*
R2	A	0.63 [IQR 0.59 0.69]	2.62 [95% CI 0.81 8.53]	0.19
R2	B	0.64 [IQR 0.58 0.71]	2.62 [95% CI 0.81 8.53]	0.19
R2	C	0.61 [IQR 0.57 0.68]	1.62 [95% CI 0.52 5.06]	0.58
R2	D	0.64 [IQR 0.58 0.70]	1.22 [95% CI 0.39 3.82]	0.95
R5	A	0.61 [IQR 0.55 0.67]	2.46 [95% CI 0.76 7.95]	0.23
R5	B	0.64 [IQR 0.55 0.72]	3.29 [95% CI 1.03 10.55]	0.09
R5	C	0.61 [IQR 0.54 0.67]	2.49 [95% CI 0.77 8.03]	0.22
R5	D	0.60 [IQR 0.56 0.68]	3.36 [95% CI 1.05 10.77]	0.08

C-index: Harrel's concordance index; HR: hazard ratio

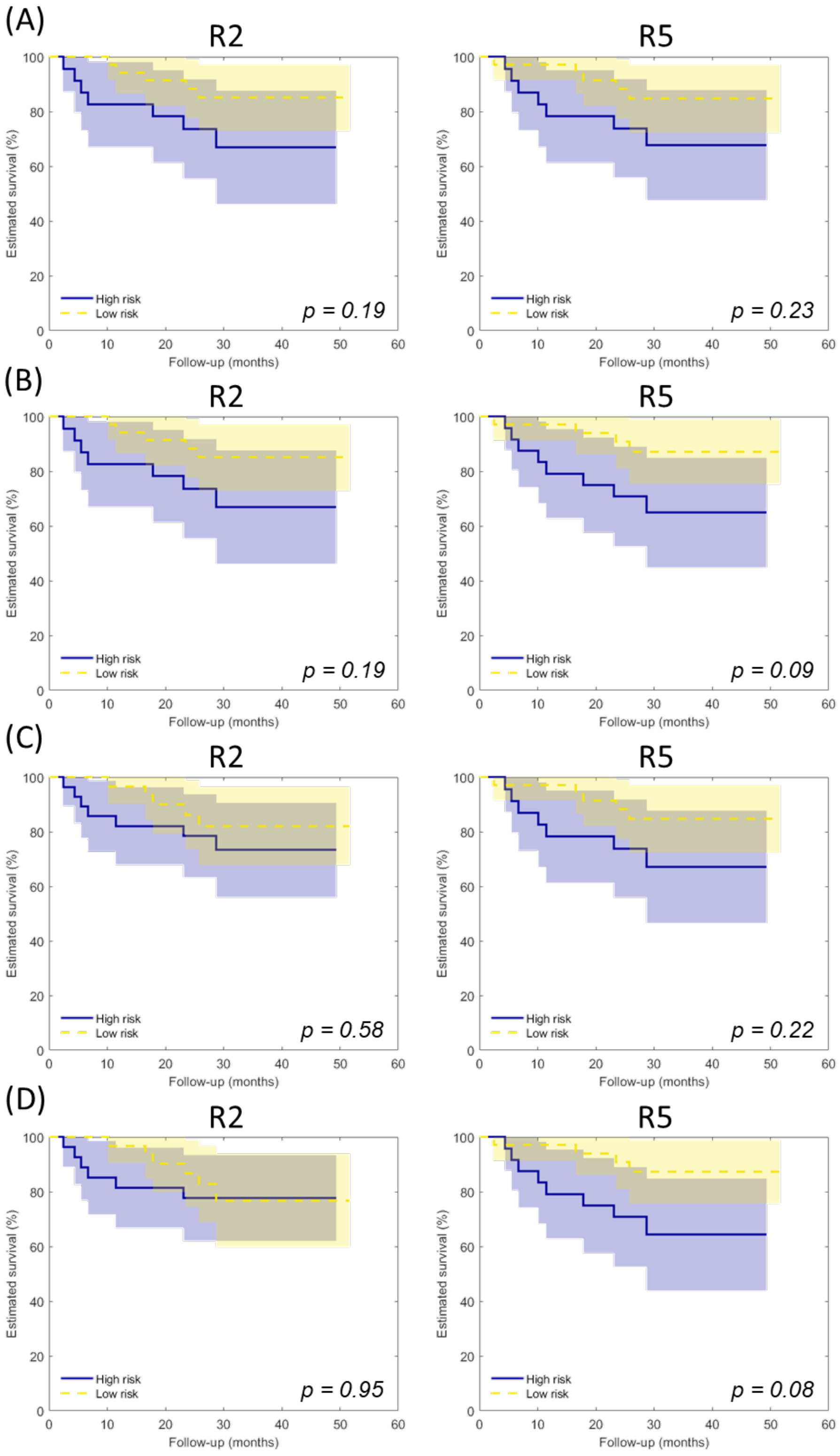

Supplementary Figure 1 Kaplan-Meier curves for the radiomic signatures R2 and R5 at different image preprocessing methods (A to D), as listed in the Supplementary Table 1.
